# Abdominal aortic aneurysm: A case report

**DOI:** 10.3892/mi.2023.103

**Published:** 2023-08-28

**Authors:** Arzu Ayraler

**Affiliations:** Department of Family Medicine, Training and Research Hospital, Giresun University, Giresun 28200, Turkey

**Keywords:** abdominal aortic aneurysm, prostatic hypertrophy, obesity

## Abstract

Abdominal aortic aneurysm (AAA) is a medical condition characterized by abnormal enlargement or the ballooning of the aorta, the largest blood vessel in the human body, in the abdomen. AAA usually develops slowly and asymptomatically and becomes a potentially life-threatening condition if left untreated. Although the exact cause of AAA is not always clear, risk factors such as age, sex, smoking, hypertension, and family history may increase the likelihood of developing AAA. It is essential to manage and prevent AAA rupture, which can lead to severe internal bleeding and pose a serious risk to a person's health if not diagnosed in a timely manner and appropriate medical attention. Awareness, early diagnosis and appropriate medical care are critical factors when addressing this condition, providing a glimpse into the complex and critical nature of AAA. The present study describes the case of a patient who applied to the family medicine unit with hypertension and dysuria, and was diagnosed with AAA incidentally.

## Introduction

Abdominal aortic aneurysm (AAA) is a critical public health concern with a prevalence ranging from 12.5 in males to 5.2% in females (1). Aneurysm rupture is associated with high morbidity and mortality rates, while can be prevented by early diagnosis and appropriate intervention (2) An aneurysm, a localized permanent and irreversible dilatation of the artery, is most common in males >65 years of age and is associated with a number of factors, including smoking, genetic factors, positive family history, a lack of regular exercise and malnutrition (3). If left untreated, the risk of mortality from continued dilation and thinning of the vessel wall is 80 to 90% (4). The optimal screening method for AAA is ultrasonography, which is inexpensive, accurate, safe, rapid, non-invasive and cost-effective (1).

## Case report

### Patient information

A 78-year-old male patient presented to the Giresun University Training and Research Hospital Family Medicine Unit (Giresun, Turkey) with symptoms of frequent urination and a burning sensation while urinating, and to have his blood pressure medications prescribed.

### Clinical findings

His medical history included known cardiovascular disease, including coronary artery disease with previous myocardial infarction and a three-vessel coronary artery bypass graft. He also had a history of 41 pack years of tobacco use, hypertension, obesity (body mass index, 29.22 kg/m^2^) and benign prostatic hypertrophy. There was no known history of aortic aneurysm. His vital signs were stable. The patient's blood pressure was 130/86 mmHg, his pulse rate was 54/min, his respiratory rate was 16/min, his oxygen saturation was 97% in room air and his body temperature was 36.5˚C. The results of the physical examination revealed natural lung auscultation, a regular heart rate and rhythm and an obese abdomen with distension; no hepatosplenomegaly was palpated. The patient had mild palpation discomfort in the abdominal region and complaints of hematuria.

The laboratory results were generally normal. The urea level was 38 mg/dl (16.6-48.5), the white blood cell count was 7.11x10^3^/l and the hematocrit was 35%. The patient was diagnosed with benign prostatic hyperplasia and a complete urinalysis and upper urinary tract ultrasound were performed according to his complaints.

### Diagnostic assessment

**Figure 1:**
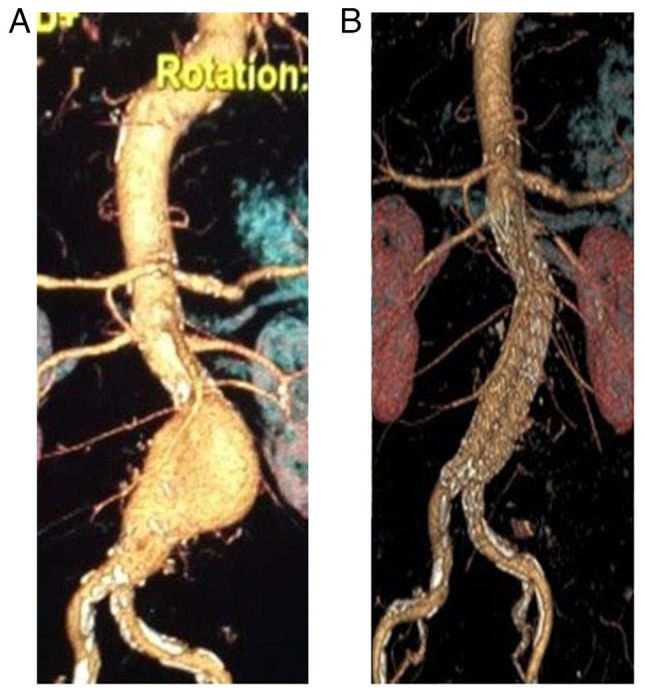
Computed tomography illustrating the abdominal aortic aneurysm (A) before and (B) post-treatment.

The diameter of the abdominal aorta at the infrarenal level was measured as 7.5 cm. A contrast-enhanced abdominal computerized tomography scan was performed for further investigation and this revealed an aneurysmatic dilatation of the abdominal aorta beginning inferior to the level of the renal arteries and continuing to the iliac bifurcation, measuring 87 mm at its widest point (Fig. 1A).

### Therapeutic intervention

Endovascular abdominal aortic aneurysm repair (EVAR) was planned under emergency conditions and an endovascular stent graft was applied to the patient with an aneurysm diameter >87 mm. EVAR is a method used to repair abdominal aortic aneurysms (4). Treatment with angiotensin 2 receptor blockers (1 per day/32 mg), acetylsalicylic acid (1 per day/100 mg) and clopidogrel (1 per day/75 mg) was commenced. The patient's general condition was good and his vital signs were normal. His blood pressure was measured as 140/60 mmHg. After 3 months, the diameter of the aortic aneurysm decreased to 70 mm in the follow-up with computed tomography (Fig. 1B). Lifestyle changes, such as quitting smoking, exercise and a healthy diet were recommended. The patient is currently being followed-up routinely by the Giresun University Training and Research Hospital.

## Discussion

AAA is defined as a permanent dilatation of the abdominal aorta that is >3 cm in diameter and usually remains asymptomatic until rupture (1). The rupture of an AAA is usually fatal, with 25% of patients succumbing before presenting to emergency services (2). Therefore, it is valuable to screen for AAA in male patients >65 years of age with ultrasonography, which is a simple and non-invasive method, taking into account the atherosclerotic risk factor. The aim of the present study was to contribute to future research on the role of common risk factors such as hypertension, obesity, family history of AAA and to address AAA screening to target the population at high risk. It is recommended that males >66 years of age be informed about the AAA screening program (5). The Society for Vascular Surgery clinical practice guidelines recommends ultrasound screening for AAA in all males >65 years of age (6). The American College of Preventive Medicine recommends 1-time screening in males 65 to 75 years of age who have ever been smokers; it does not recommend routine screening in females (7). The American College of Cardiology and the American Heart Association jointly recommend 1-time screening for AAA with a physical examination and an ultrasonography in males aged 65 to 75 years who have ever been smokers or in males ≥60 years who are the sibling or offspring of an individual who has suffered an AAA (8). Appropriate screening programs for AAA reduce the mortality rates and are cost-effective (9).

In conclusion, with the continuous increase in life expectancy, the importance of preventive health care is increasing. Preventive health care is a fundamental aspect of modern medicine and should be encouraged and supported in a health care system that deals with the individual, not merely the disease itself, even if the individual is healthy. Although AAA is associated with high mortality rates when it ruptures, it is a preventable disease if diagnosed at an early stage (2). Preventive measures need to be promoted in a healthcare system that takes care of the individual, even if the individual is in good health (1). It is recommended that the screening of individuals >65 years of age for AAA be performed with ultrasonography, which is an inexpensive, accurate, safe, rapid, non-invasive, reproducible and cost-effective method.

## Data Availability

The datasets used and/or analyzed in the current study are available from the corresponding author upon reasonable request.
